# Gender as a moderator of the relationship between workplace violence and burnout among healthcare professionals

**DOI:** 10.3389/fpubh.2026.1948879

**Published:** 2026-09-15

**Authors:** Yong-Hsin Chen, Ming-Yu Hsieh, Ching-Wen Yang, Gwo-Ping Jong, Kuang-Chen Hung

**Affiliations:** 1 Department of Public Health, Chung Shan Medical University, Taichung, Taiwan; 2 Department of Occupational Safety and Health, Chung Shan Medical University Hospital, Taichung, Taiwan; 3 School of Medicine, Chung Shan Medical University, Taichung, Taiwan; 4 Division of Pediatric Surgery, Department of Surgery, Chung Shan Medical University Hospital, Taichung, Taiwan; 5 Center for Evidence-Based Medicine, Department of Medical Education, Chung Shan Medical University Hospital, Taichung, Taiwan; 6 Department of Internal Medicine, Chung Shan Medical University Hospital, Taichung, Taiwan; 7 Department of Surgery, Kaohsiung Veterans General Hospital, Kaohsiung, Taiwan; 8 College of Nursing, Meiho University, Pingtung, Taiwan; 9 School of Medicine, National Defense Medical University, Taipei, Taiwan

**Keywords:** alcohol use, gender, psychological violence, workplace violence, work-related burnout

## Abstract

**Background:**

Work-related burnout (WB) is a pervasive issue among healthcare workers often exacerbated by workplace violence or aggression (WVA). Although gender differences in burnout are well-documented, the moderating role of gender in the relationship between WVA and WB remains understudied. This study aims to examine the associations between WVA, alcohol use, and WB among Taiwanese healthcare professionals by specifically investigating whether gender moderates these relationships.

**Methods:**

Secondary data were analyzed from a cross-sectional survey conducted in March 2025 among 907 frontline healthcare workers at a university-affiliated hospital in Taichung, Taiwan. WB was assessed using the Copenhagen Burnout Inventory. Multiple linear regression models with interaction terms (female × WVA; female × alcohol use) were constructed, with empirical 95% confidence intervals derived from a nonparametric percentile bootstrap (5,000 replications) to confirm the robustness of the estimates.

**Results:**

WVA was significantly associated with increased WB (B = 10.61, *p* < 0.001). A significant negative interaction between female gender and WVA (B = −10.08, 95% CI: −17.29 to −2.90) indicated that the magnitude of burnout increase associated with WVA was significantly larger among male workers than female workers. Conversely, an exploratory crossover interaction was observed for alcohol use, where drinking was associated with higher burnout in females but showed a null or non-significant association in males. Other key predictors included psychological violence, frequent overtime, and irregular shift work.

**Conclusion:**

Gender significantly moderates the impact of occupational stressors on burnout. Preliminary evidence suggests that male workers may exhibit greater sensitivity to the specific stressor of WVA, whereas the association between alcohol use and burnout was observed predominantly among female workers, highlighting potential sex-specific coping patterns that warrant further longitudinal exploration. These findings underscore the necessity for gender-responsive interventions, such as emotional support for men and substance use tracking for women, to effectively reduce burnout.

## Introduction

Work-related burnout (WB) is a psychological syndrome marked by emotional exhaustion, depersonalization, and diminished sense of personal accomplishment resulting from prolonged occupational stress (1). It is particularly prevalent among healthcare workers, where emotional labor (2), shift rotations (3), long working hours (4), and interpersonal demands (5) are frequent. Recent studies have reported rising burnout rates in the healthcare sector. For example, a 2025 JAMA Network Open study of over 10,000 healthcare workers across 140 medical centers found that the prevalence of WB increased from 30.4% in 2018 to 39.8% in 2022 and then declined slightly to 35.4% in 2023 (6). Similarly, a CDC report indicated that as of 2022, 46% of U.S. healthcare workers reported frequent symptoms of burnout, underscoring the ongoing strain imposed by pandemic-related working conditions (7).

Several occupational and personal factors contribute to burnout in the healthcare settings. Among organizational determinants, overtime, shift work, low job control, and role conflict have been consistently linked to an elevated risk of WB (8, 9). Additionally, family-related stressors—including marital status, parenting responsibilities, and lack of social support—may further intensify this risk (10). These cumulative pressures undermine individual wellbeing and adversely affect patient safety, workforce retention, and healthcare system performance.

Among various occupational stressors, workplace violence or aggression (WVA) has emerged as a critical healthcare concern. According to the Occupational Safety and Health Administration (OSHA), WVA encompasses physical assaults, verbal abuse, threats, and other aggressive behaviors commonly encountered in clinical settings—often perpetrated by patients, family members, or even colleagues (11). Its prevalence is alarmingly high; the WHO reported that over half of healthcare workers worldwide have experienced some form of WVA during their careers, especially in high-risk departments such as emergency and critical care (12). Additionally, a regional survey reported that 58% of registered nurses in the South-East Asia and Western Pacific Regions had experienced WVA (13). Exposure to WVA has also been linked to anxiety, depression, posttraumatic stress symptoms, and elevated burnout levels (14).

In 2020, Taiwan had approximately 180,000 registered nursing professionals, of whom 97.5% were women and 2.5% were men (15). This pronounced gender imbalance underscores the importance of examining gender-specific responses to workplace stressors in healthcare settings.

Gender may moderate the psychological impact of occupational stressors, such as WVA. Biopsychosocial frameworks posit that biological, psychological, and sociocultural differences between men and women shape distinct coping responses (16). Generally, women are more likely to employ emotion-focused coping and seek social support, whereas men are more likely to adopt problem-focused strategies or engage in emotional suppression (17). In the context of workplace aggression, these gendered coping styles and perceptions may shape how men and women experience and respond to WVA. However, few empirical studies have tested whether gender moderates the relationship between WVA and burnout, particularly in healthcare settings.

Accordingly, two hypotheses were formulated to guide this study. Hypothesis 1 posits that exposure to WVA increases the risk of burnout, whereas Hypothesis 2 proposes that gender moderates this relationship. These hypotheses reflect theoretical perspectives suggesting that gendered coping patterns and sociocultural norms may shape differential responses to workplace adversity. The conceptual framework of the study, which hypothesizes that gender moderates the relationship between WVA and WB, is illustrated in Figure 1.

**Figure 1:**
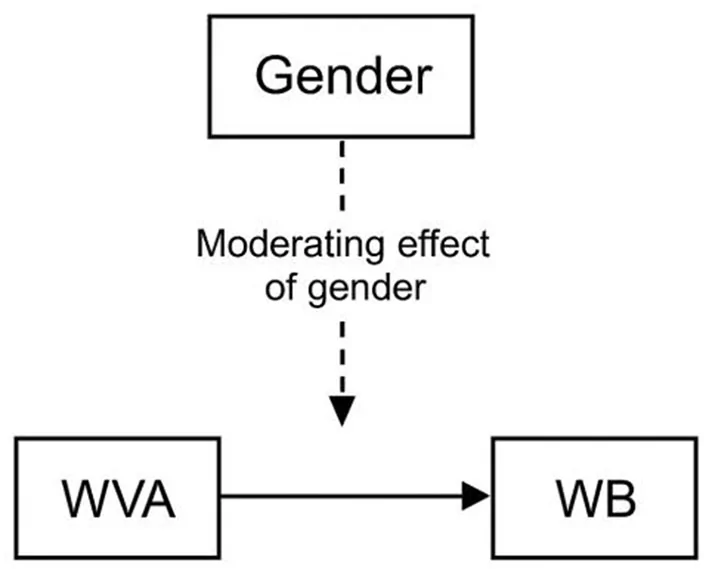
Conceptual framework of the study.

## Methods

This study used a cross-sectional observational design, drawing on secondary data collected in March 2025 from a teaching hospital affiliated with a medical university in Taichung, Taiwan. The survey was distributed via QR code links embedded in email invitations sent through the hospital's internal communication system. The electronic survey platform required all mandatory items to be completed before final submission, resulting in zero item-level missing data among the submitted responses. Of the hospital's 3,217 employees, a total of 1,298 responses were received. Based on self-reported information in the questionnaire, 912 respondents were identified as frontline workers with direct contact with patients or their families. Extreme values were screened and addressed by excluding non-representative outliers using the interquartile range (IQR) method to prevent influential cases from distorting ordinary least squares (OLS) estimations; the specific criteria and procedures are described in the Statistical Analysis section. After data cleaning, 907 valid cases remained and were included in the regression analysis. The dataset was anonymized, and the study protocol was approved by the hospital's Institutional Review Board (IRB No.: CS2-25081).

## Measurements

### Work-related burnout

Work-related burnout (WB) was evaluated using the WB subscale of the Copenhagen Burnout Inventory (CBI), adapted from the official Chinese version of the Work Overload Assessment Questionnaire published by the Taiwan Ministry of Labor (18). The CBI comprises three distinct subscales: personal burnout, WB, and client-related burnout. In accordance with the original instrument developers, each subscale possesses distinct psychometric properties and can be administered independently based on the specific research focus (19). Therefore, only the WB subscale was utilized in the present study to specifically capture physical and psychological exhaustion stemming from occupational stressors such as workplace violence. The subscale consists of seven items: (1) Is your work emotionally exhausting? (2) Do you feel burnt out because of your work? (3) Does your work frustrate you? (4) Do you feel worn out at the end of the working day? (5) Are you exhausted in the morning at the thought of another day at work? (6) Do you feel that every working hour is tiring for you? (7) Do you have enough energy for family and friends during leisure time? (reverse scored). These responses were converted to a 0–100 scoring system as follows: for Items 1–6, always = 100, often = 75, sometimes = 50, seldom = 25, and never = 0. Item 7, which is negatively worded, was reverse coded using the same scale (always = 0 to never/rarely = 100). The final WB score was calculated as the average of the seven item scores, with higher scores indicating greater levels of work-related exhaustion.

### Workplace violence or aggression (workplace unlawful harm) and perceived violence types

Workplace violence or aggression (WVA) was evaluated using two independent, non-conditional self-reported items administered to all participants. In accordance with the official Taiwanese Ministry of Labor guidelines (Directive for Preventing Unlawful Harm When Performing Work Duties) (20), WVA in this study operationally captures workplace unlawful harm, defined as “unlawful physical or psychological harm caused to an employee by service recipients, third parties, or workplace internal personnel (employers, supervisors, or colleagues) during the execution of work duties through verbal abuse, written statements, physical actions, electronic communications, internet communications, or other forms of violence or inappropriate behavior.”

Based on the official directive above, participants reported the overall frequency of formal workplace unlawful harm experiences over the past year on a five-point Likert scale (always, often, sometimes, seldom, and never).

To capture the broader landscape of daily clinical stress and occupational risk perception, all frontline healthcare workers (*N* = 907) were separately asked to identify the predominant form of workplace violence or inappropriate behavior they perceive as most likely to be encountered in their daily work environment (categorized into verbal abuse, physical violence, psychological violence, or sexual harassment/other forms). This single-choice item captures overall occupational risk perception across the entire workforce, regardless of whether an individual formally filed or experienced an official “unlawful harm event” during the study period.

### Sociodemographic and occupational factors

The questionnaire collected information on various sociodemographic and work-related variables. Sex was coded as female or male, and participants reported their age as a continuous variable. While self-reported biological sex was captured as a binary variable, it served as an operational proxy in our analyses to examine gendered social roles and occupational stress responses. Marital status was categorized as unmarried, < 2, 2–5, 5–10, 10–20, and >20 years. Parenting status was assessed as a binary variable (yes/no).

Leisure activity with family and friends (LAFF) was measured using a 5-point Likert scale: always, often, sometimes, seldom, and never. Sleep duration was recorded using five response categories: less than 5, 5, 6, 7, and 8 h per day.

Participants provided their height and weight, which were used to calculate body mass index using the following formula: weight (kg)/height^2^ (m^2^). Alcohol use in the past month was recorded as never, sometimes, or every day. Exercise frequency was self-reported on a five-point scale: never, less than once a month, at least once a month, at least once a week, and almost every day.

Educational attainment was categorized as high school, bachelor's degree, master's degree, or PhD. Occupational roles were classified into four groups: physicians, nurses, allied health professionals, and administrative or other staff.

Overtime work frequency per month was assessed in four categories: seldom, < 45, 45–80, and >80 h. Shift work was categorized as irregular, regular, night, and day.

### Statistical analysis

Outliers in WB scores were identified and removed using the IQR method. Descriptive statistics were computed for all variables. Univariate analyses included independent *t*-tests, one-way ANOVA with *post hoc* comparisons (Duncan's multiple range test), and Pearson's correlation. Multiple linear regression models were constructed to estimate the association between WVA and WB while adjusting for confounders and to assess the interaction effect between sex and reported WVA on WB.

To evaluate the robustness of the regression estimates against potential non-normality and heteroscedasticity, a nonparametric percentile bootstrap method with 5,000 replications was applied. This resampling approach provides empirical confidence intervals without relying on standard distributional assumptions, although it does not alter the inherent sample constraints of sparse subgroup cells. It is a suitable approach for validating coefficient stability and the inference robustness in the presence of imbalanced or heterogeneous data structures (21). Variance inflation factors (VIFs) were examined to assess multicollinearity. The analysis was conducted using SAS Enterprise Guide 7.1 software (SAS Institute Inc., Cary, NC, USA), with significance defined as *p* < 0.05.

## Results

### Descriptive comparison of WB

In the study sample, the seven-item WB subscale demonstrated high internal consistency (Cronbach's alpha = 0.89). Table 1 compares the raw dataset with the dataset after outlier removal using the IQR method. Five cases were excluded. The mean, median, and quartiles remained consistent across both datasets (mean: 37.08 vs. 36.77; median: 35.71), while skewness and kurtosis decreased from 0.24 to 0.12 and from 0.30 to 0.04, respectively. These reductions indicate improved normality. Notably, both skewness (0.12) and kurtosis (0.04) fell within the acceptable ±1 range, suggesting an approximately normal distribution and supporting the use of linear regression (22).

**Table 1:** Comparison between raw data (I) and data after outlier removal using the interquartile range (IQR) method (II) for WB.

Model	*N*	Mean ±SD	Q1	Median	Q3	Kurtosis	Skewness
I	912	37.08 ± 17.72	25.00	35.71	50.00	0.30	0.24
II	907	36.77 ± 17.26	25.00	35.71	50.00	0.04	0.12

Model I: raw data; Model II: data after outlier removal using the IQR method.

### Univariate associations with WB

Table 2 presents the associations between WB and demographic, behavioral, and occupational variables. Among the continuous variables, WB scores were significantly and negatively associated with age (*r* = −0.18, *p* < 0.001) and work experience (*r* = −0.13, *p* < 0.001) and significantly differed across categories of daily sleep duration (*p* = 0.036) and exercise frequency (*p* < 0.001).

**Table 2:** Relationship between WB and survey variables.

Variable	*N*	Work-related Burnout	
		Mean ±SD/*r*	*p*-Value
Age	907	−0.18	< 0.001
Sex			
Female	772	37.38 ± 16.95	0.011
Male	135	33.28 ± 18.65	
Marital tenure			
Unmarried	434	39.55 ± 17.07^ab^	< 0.001
< 2 years	22	40.58 ± 18.33^a^	
2–5 years	60	35.89 ± 17.67^ab^	
5–10 years	91	39.44 ± 17.31^ab^	
10–20 years	168	33.80 ± 16.40^bc^	
>20 years	132	29.30 ± 15.92^c^	
Parenting state			
Yes	371	34.39 ± 17.00	0.001
No	536	38.41 ± 17.27	
Leisure activity with family and friends in holiday (LAFF)			
Always (100 points)	67	30.54 ± 17.97^a^	0.001
Often (75 points)	251	35.20 ± 17.71^ab^	
Sometime (50 points)	451	37.46 ± 17.31^ab^	
Seldom (25 points)	127	39.90 ± 14.97^bc^	
Never (0 points)	11	45.46 ± 12.69^c^	
Daily sleep duration			
< 5 h	33	44.70 ± 19.15^a^	0.036
5–6 h	360	37.60 ± 17.04^b^	
6–7 h	382	35.56 ± 17.37^b^	
7–8 h	108	36.34 ± 15.54^b^	
>8 h	24	34.52 ± 21.08^b^	
Body mass index	907	−0.02	0.482
Alcohol use in the past month			
Every day	3	25.00 ± 22.30	0.053
Sometime	364	38.29 ± 17.01	
Never	540	35.81 ± 17.35	
Exercise frequency			
Almost every day	65	32.80 ± 17.60^c^	< 0.001
At least once a week	391	34.75 ± 17.95^c^	
At least once a month	165	36.99 ± 16.00^bc^	
Less than once a month	215	39.67 ± 15.56^ab^	
Never	71	42.20 ± 18.59^a^	
Work experience	907	−0.13	< 0.001
Frequency of workplace violence or aggression			
Often/sometime	28	53.57 ± 13.25^a^	< 0.001
Seldom	150	46.19 ± 16.13^b^	
Never	729	34.20 ± 16.61^c^	
Violence type			
Physical violence	140	38.34 ± 17.55^b^	< 0.001
Psychological violence	102	44.22 ± 17.43^a^	
Verbal abuse	611	35.63 ± 16.88^bc^	
Other	54	31.48 ± 16.45^c^	
Education degree			
PhD degree	28	33.55 ± 20.92^ab^	0.041
Master's degree	118	34.63 ± 15.96^ab^	
Bachelor's degree	747	37.41 ± 17.27^a^	
High school education	14	27.04 ± 16.14^b^	
Professional field			
Physician	54	33.53 ± 19.07^b^	< 0.001
Nurse	538	40.73 ± 16.19^a^	
Allied health professionals	128	30.22 ± 16.08^b^	
Administrative staff or other staff members	187	30.79 ± 17.34^b^	
Frequent monthly overtime			
>80 h	3	45.24 ± 13.52^a^	< 0.001
45–80 h	27	45.90 ± 17.90^a^	
< 45 h	352	42.44 ± 16.39^a^	
Seldom	525	32.44 ± 16.54^a^	
Shift work type			
Irregular shift work	148	43.22 ± 15.99^a^	< 0.001
Regular shift work	131	37.79 ± 16.21^b^	
Night work	98	40.85 ± 16.49^ab^	
Day work	530	33.96 ± 17.37^c^	

SD, standard deviation; r, Pearson correlation coefficient; Groups labeled with different letters (^a, b, c^) differ significantly from each other based on *post hoc* comparisons (Duncan's multiple range test).

Regarding categorical variables, participants with parenting responsibilities reported lower WB than those without children (34.39 ± 17.00 vs. 38.41 ± 17.27, *p* = 0.001). Females reported significantly higher WB scores than males (37.38 ± 16.95 vs. 33.28 ± 18.65, *p* = 0.011). To address whether this gender discrepancy in burnout was confounded by differential exposure to workplace violence, an additional analysis was conducted. No statistically significant difference in WVA exposure frequency was observed between male (5.00 ± 12.14) and female (5.83 ± 12.35) healthcare workers (*p* = 0.471), indicating that WVA exposure was comparable across genders.

Significant differences in burnout were observed across marital tenure groups (*p* < 0.001), with individuals married for < 2 years exhibiting the highest scores (40.58 ± 18.33) and those married for over 20 years the lowest (29.30 ± 15.92). WB scores increased as LAFF decreased (*p* = 0.001), with the “never” group reaching a mean of 45.46 ± 12.69 compared to 30.54 ± 17.97 in the “always” group.

A similar pattern was observed for the occupational conditions. Participants with frequent overtime (45–80 h/month) reported the highest WB (45.90 ± 17.90), whereas those who seldom worked overtime reported lower WB (32.44 ± 16.54; *p* < 0.001). WB also varied significantly by shift type (*p* < 0.001), with irregular shift workers experiencing the highest scores (43.22 ± 15.99), followed by night workers (40.85 ± 16.49), regular shift workers (37.79 ± 16.21), and day workers (33.96 ± 17.37).

Frequent exposure to WVA was significantly associated with increased burnout levels (*p* < 0.001). To address extreme sample size imbalances in the initial 5-point scale (i.e., *n* = 0 for “Always” and *n* = 1 for “Often”), response options were consolidated into three operationally meaningful categories prior to statistical analysis: Often/Sometimes (*n* = 28), Seldom (*n* = 150), and Never (*n* = 729). *Post-hoc* pairwise comparisons using Duncan's test revealed a significant dose-response relationship, with participants reporting exposure “often or sometimes” experiencing the highest WB score (53.57 ± 13.25), followed by those reporting “seldom” (46.19 ± 16.13), and those with no reported WVA (“never”) exhibiting the lowest WB score (34.20 ± 16.61).

Among the types of violence experienced, psychological violence was associated with the highest WB scores (44.22 ± 17.43), followed by physical violence (38.34 ± 17.55), verbal abuse (35.63 ± 16.88), and other types (31.48 ± 16.45).

*Post hoc* analyses confirmed that elevated WB scores were particularly concentrated among individuals who experienced psychological violence, those with high overtime workloads, and those with minimal LAFF.

### Regression models for WB

Table 3 presents two linear regression models that examine the association between reported WVA, demographic and occupational variables, and WB.

**Table 3:** Unadjusted and adjusted linear regression models (models A and B) for WB.

Main effect	Model A		Model B		
	B	*p*-Value	B	*p*-Value	VIF
Reported WVA^a^	13.07	< 0.001	10.53	< 0.001	1.100
Female^b^	4.10	0.011	−0.08	0.959	1.358
Confounder					
Age	−0.28	< 0.001	−0.12	0.152	3.374
Marital tenure < 10 years^c^	−1.57	0.32	−0.11	0.958	2.266
Marital tenure 10–20 years^c^	−5.80	< 0.001	−1.81	0.446	3.435
Marital tenure >20 years^c^	−10.30	< 0.001	−4.85	0.080	3.808
Raising children^d^	−4.03	< 0.001	0.15	0.941	4.139
LAFF	−0.12	< 0.001	−0.05	0.075	1.128
Sleep duration < 5 h^e^	8.69	0.010	5.76	0.058	1.306
Sleep duration 5–6 h^e^	1.59	0.365	1.02	0.518	2.409
Sleep duration 6–7 h^e^	−0.46	0.793	1.21	0.435	2.373
Exercise almost daily^f^	−7.50	0.002	−3.20	0.139	1.256
Exercise at least once a week^f^	−5.55	< 0.001	−3.51	0.005	1.537
Exercise at least once a month^f^	−3.31	0.048	−3.39	0.024	1.370
Work experience	−0.22	< 0.001	−0.01	0.952	2.848
Physical violence^g^	3.05	0.054	1.05	0.462	1.086
Psychological violence^g^	8.93	< 0.001	6.81	< 0.001	1.074
PhD/master's degree^h^	−2.80	0.073	1.39	0.357	1.255
Physician^i^	2.75	0.285	−0.38	0.883	1.533
Nurse^i^	9.94	< 0.001	5.41	< 0.001	1.820
Technical staff^i^	−0.57	0.765	−1.29	0.472	1.589
Ever overtime^j^	10.27	< 0.001	6.81	< 0.001	1.148
Irregular shift work^k^	9.26	< 0.001	3.32	0.029	1.270
Regular shift work^k^	3.83	0.020	0.80	0.604	1.187
Night work^k^	6.89	< 0.001	3.30	0.066	1.257

Model A: unadjusted model including only reported WVA and gender (confounders evaluated via separate unadjusted bivariate models).

Model B: adjusted model including all covariates listed in the table.

^*a*^Never reported WVA.

^*b*^Male.

^*c*^Unmarried.

^*d*^Without raising children.

^*e*^Sleep duration >7 h (collapsed from 7–8 and >8 h categories to establish a stable healthy reference group).

^*f*^Exercise less than once a month or never.

^*g*^Verbal violence or other.

^*h*^Bachelor's degree or other.

^*i*^Administrative staff or other.

^*j*^Seldom work overtime.

^*k*^Day shift work.

B, linear regression coefficient; *p, p*-value; VIF, variance inflation factor.

Model A is the unadjusted model, including only WVA and gender (with candidate confounders evaluated via separate unadjusted bivariate regressions). Model B is the adjusted model that incorporates all pre-specified demographic, occupational, and lifestyle covariates simultaneously. In Model A, both reported WVA (B = 13.07, *p* < 0.001) and female gender (B = 4.10, *p* = 0.011) were significantly associated with higher WB scores.

After adjustment in Model B, the reported WVA remained a significant predictor (B = 10.53, *p* < 0.001), thereby supporting Hypothesis 1. However, the effect of gender became nonsignificant (B = −0.08, *p* = 0.959), suggesting that its influence may be confounded by occupational and lifestyle-related factors.

Several covariates remained significant in Model B, including psychological violence (B = 6.81, *p* < 0.001), the nursing profession (B = 5.41, *p* < 0.001), overtime (B = 6.81, *p* < 0.001), and irregular shift work (B = 3.32, *p* = 0.029). All VIFs were below 5, indicating no serious multicollinearity.

The adjusted *R*-squared value of Model B was 0.251, indicating that the model explained approximately 25.1% of the variance in WB scores. The Durbin–Watson statistic was 2.033, indicating no evidence of first-order autocorrelation. Model residuals exhibited minimal skewness (0.15) and kurtosis (0.16). All tests of normality confirmed that residuals followed an approximately normal distribution: Shapiro–Wilk test (W = 0.9975, *p* = 0.1781), Kolmogorov–Smirnov (D = 0.0223, *p* > 0.15), Cramér–von Mises (W^2^ = 0.0637, *p* > 0.25), and Anderson–Darling (A^2^ = 0.435, *p* > 0.25).

### Interaction between gender, WVA, and alcohol use on WB

Two sets of linear regression models were constructed to examine whether gender moderates the relationship between two key stressors—reported WVA and alcohol use in the past month—and WB (Table 4). Model C refers to a conventional multiple linear regression adjusted for all covariates listed in Table 3, whereas Model D presents the corresponding bootstrap-adjusted results based on 5,000 replications.

**Table 4:** Linear regression model examining the interaction of female gender and reported WVA/alcohol use in the past month on WB.

Survey variable	WB			
	Model C		Model D	
	B	95% CI	B	95% CI
Female	1.81	−1.60, 5.22	1.78	−1.69, 5.22
Reported WVA	19.03	12.06, 26.01	19.33	12.72, 26.07
Female × reported WVA	−9.90	−17.34, −2.47	−10.19	−17.30, −3.00
Adjusted *R*^2^	0.26		—	
Female	−4.12	−8.39, 0.12	−4.06	−8.51, 0.41
Alcohol use in the past month	−5.37	−10.46, −0.25	−5.14	−10.24, −0.04
Female × alcohol use in the past month	8.42	2.86, 13.99	8.18	2.63, 13.79
Adjusted *R*^2^	0.26			

Model C: multiple linear regression adjusted for all covariates listed in Table 3 (model B). Model D: Bootstrap-adjusted regression model (5,000 replications), adjusted for the same covariates as in Model C. Reference groups: male (female), no reported WVA (reported WVA), and no alcohol use in past month (alcohol use in past month). B, linear regression coefficient; 95% CI, 95% confidence interval.

The interaction term Female × Reported WVA was statistically significant in both Model C (B = −9.90, 95% CI: −17.34 to −2.47) and Model D (B = −10.19, 95% CI: −17.30 to −3.00), supporting Hypothesis 2. Although reported WVA was associated with a significant increase in WB (B ≈ 19), this effect was significantly attenuated among females, suggesting gender's potential buffering role.

In contrast, the interaction term Female × Alcohol use in the past month was also significant in both models (Model C: B = 8.42, 95% CI: 2.86–13.99; Model D: B = 8.06, 95% CI: 2.74–13.62). In this case, alcohol use was associated with higher burnout among women yet slightly lower predicted scores among men.

As illustrated in Figure 2A, WB increased more steeply among males exposed to WVA, whereas the effect was less pronounced among females. Figure 2B reveals a crossover interaction, where burnout scores rose in female alcohol users while declining modestly among male alcohol users.

**Figure 2:**
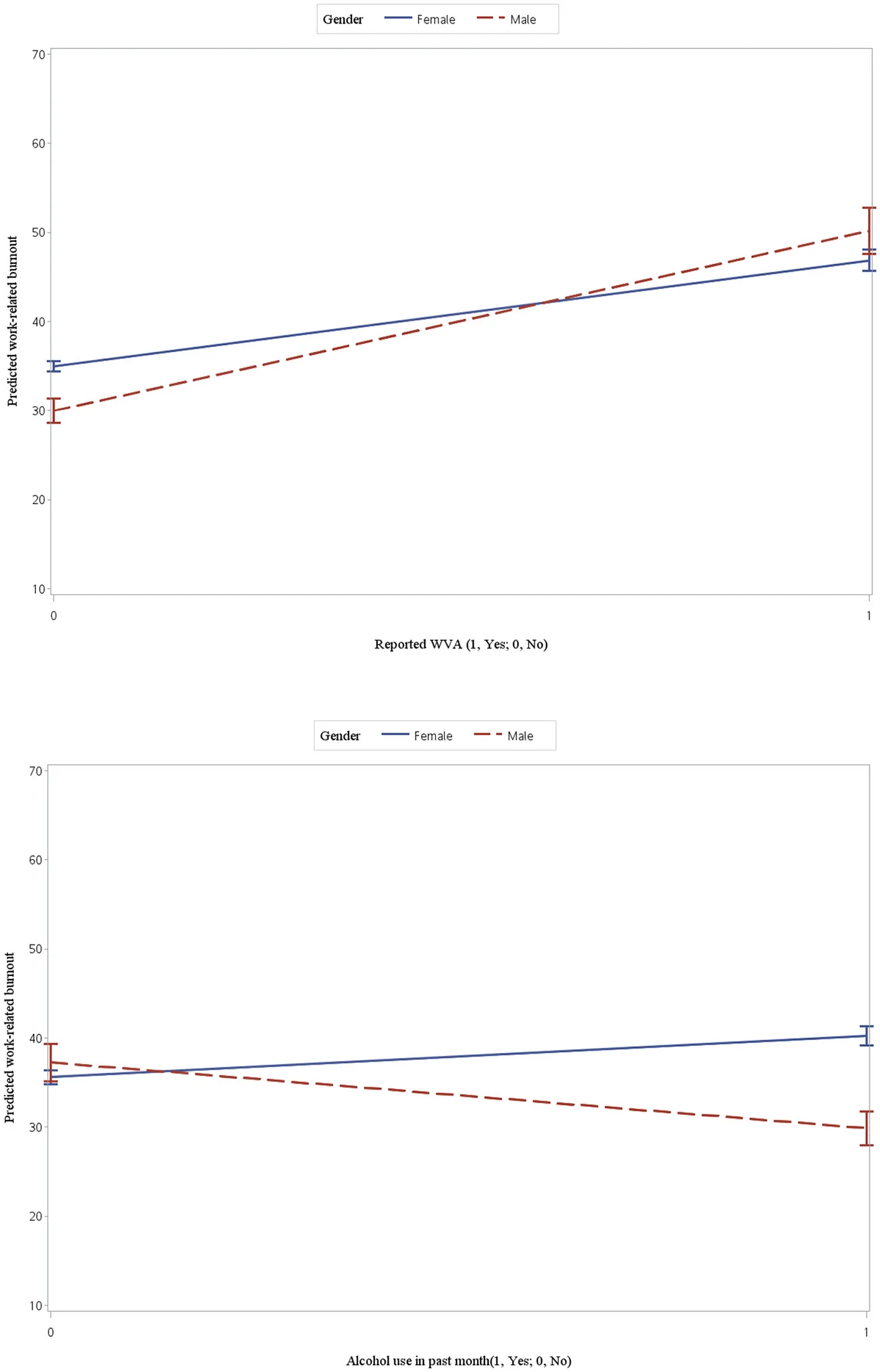
**(A)** Interaction of reported WVA and gender on WB. **(B)** Interaction between gender and alcohol use on predicted WB.

Taken together, these opposing interaction effects—attenuation in the WVA model and amplification in the alcohol use model—underscore the context-dependent nature of gender's moderating role in stress–burnout dynamics. These findings may reflect distinct coping patterns or sociocultural mechanisms underlying the gendered responses to different stressors.

## Discussion

1. Relationship between workplace violence and WB

Consistent with the literature, our findings demonstrate a significant positive association between WVA and WB among healthcare workers. This aligns with a growing body of evidence identifying WVA as a critical occupational stressor that significantly increases the risk of burnout in this population. A recent systematic review and meta-analysis showed that nurses who experienced WVA were more than twice as likely to experience burnout as their unexposed peers (23). Similarly, a large-scale study using secondary data found that workplace violence significantly affected the psychological health of medical-surgical nurses, with burnout mediating the impact (24). Workplace violence not only has mental health consequences but is also associated with reduced care quality and increased risk of medical errors (25). These findings underscore the importance of institutional policies that actively prevent and address WVA to reduce burnout and protect workforce sustainability.

2. Model robustness and consistency with previous evidence

We ensured the credibility of our findings by assessing the robustness of the adjusted regression model (Model B) using multicollinearity diagnostics, residual analysis, and autocorrelation checks—all of which indicated an acceptable model fit. Additionally, we applied a nonparametric percentile bootstrap procedure (Model D) to evaluate the empirical 95% confidence intervals for the interaction terms, thereby accounting for potential non-normality and heteroscedasticity in the data. This approach reinforced the reliability of the gender moderation effect and aligned with the recommended practices for inference under imbalanced or heterogeneous sample conditions (26).

Furthermore, several independent predictors remained significant in Model B—including psychological violence, frequent overtime, irregular shift work, and the nursing profession—which have also been corroborated by previous research. For instance, long working hours and shift rotations have been repeatedly linked to emotional exhaustion and circadian rhythm disruption (27). Exposure to psychological aggression also undermines a sense of personal accomplishment and increases depersonalization, exacerbating burnout (28). The alignment of our results with the literature enhances the model's external validity.

3. Mechanisms explaining gender differences in the WVA–burnout link

After confirming model validity, we examined the interaction between gender and WVA (Models C and D), with particular attention to the differential effects observed among female healthcare workers. Our findings indicate that the impact of WVA on WB is attenuated among women. We clarify this gendered moderation effect by drawing on psychological constructs and gender-specific coping frameworks that may explain women's differential burnout responses to WVA.

Emerging evidence suggests that gender plays a critical role in shaping how individuals respond to WVA, particularly in healthcare settings. Biological and psychosocial frameworks indicate that men and women tend to exhibit distinct stress–response patterns, with women more likely to adopt a “tend-and-befriend” strategy—seeking social support and fostering interpersonal relationships under stress. In contrast, men often display “fight-or-flight” behaviors, including emotional suppression or externalizing reactions, such as irritability and aggression (29). Male healthcare workers may perceive WVA as a threat to professional authority or control, potentially exacerbating burnout responses, especially when combined with limited emotional disclosure or reluctance to seek support (30). These divergent coping tendencies may influence both immediate emotional responses and long-term psychological outcomes, including burnout.

Although psychological mechanisms were not directly measured in this study, these gendered coping patterns may be partially understood through the sense of coherence (SOC) framework. SOC refers to an individual's capacity to perceive stressful events as comprehensible, manageable, and meaningful, thereby facilitating adaptive responses to adversity (31, 32). We speculate that because women are more likely to engage in “tend-and-befriend” strategies, they might draw upon stronger SOC tendencies, particularly in terms of meaningfulness and manageability. In the context of WVA, this inclination to seek social support and cultivate emotional bonds could plausibly enhance women's perceived emotional resources and reinforce their coping efficacy. Consequently, SOC might theoretically server as a psychological buffer moderating the relationship between WVA and burnout among female healthcare workers (33). Nevertheless, because SOC and coping behaviors were not psychometrically evaluated in our sample, these interpretations remain tentative hypotheses that warrant future empirical validation.

Supporting this interpretation, our supplementary analysis found that alcohol use was more strongly associated with burnout among female healthcare workers. In contrast, male burnout did not vary across drinking behaviors.

This interaction pattern suggests that when women deviate from their typical interpersonal coping style—such as turning to alcohol, an avoidant intrapersonal strategy—they may become more prone to experiencing emotional exhaustion. Alcohol use is widely recognized as an avoidant coping behavior, often used to escape rather than resolve stressors. It is associated with negative psychological outcomes when overused in high-stress contexts (34, 35).

Research indicates that women are more likely to use emotion-focused and social coping strategies. Conversely, avoidant behaviors, such as substance use, are more common—and less psychologically taxing—among men (16, 17, 36).

Altogether, these findings reinforce the notion that gendered coping styles, especially those rooted in interpersonal regulation, may protect women from burnout in situations involving interpersonal stressors, such as WVA. Conversely, maladaptive deviations from these strategies (e.g., alcohol use) may erode that psychological buffer. Therefore, the observed moderation by gender in both WVA and alcohol-related burnout pathways lends convergent support to the idea that socially conditioned coping preferences—such as the “tend-and-befriend” model—are key to shaping burnout vulnerability across different stress domains (29, 37).

These findings underscore the importance of treating gender not only as a demographic covariate but as a substantive moderator in WVA-related burnout research. Developing gender-responsive intervention strategies that acknowledge these coping differences may enhance the effectiveness of workplace violence prevention and burnout mitigation efforts in healthcare settings. For instance, interventions for male personnel could prioritize nonstigmatizing channels for emotional expression to counteract men's reluctance to seek support. Conversely, for female staff, monitoring for maladaptive shifts in coping behavior—such as increased alcohol use—could identify critical early warning signs of burnout vulnerability.

### Limitations

This study has several limitations. First, owing to the questionnaire's design constraints, we were unable to investigate the underlying psychological or social mechanisms that may contribute to the buffering effect of female gender on WB in the context of WVA. Although the analysis draws on the SOC framework to interpret the observed gender differences, no SOC-related variables were included in the survey instrument. Consequently, this interpretation remains theoretical rather than empirically validated, and it should be viewed as a hypothesis-generating insight rather than a conclusion supported by the data. Second, our sample exhibited a notable gender imbalance, with relatively few male participants. Although we used a bootstrap resampling approach to address estimation bias arising from unequal group sizes, the limited representation of male respondents may still compromise the precision and stability of the interaction effect estimates. This imbalance poses challenges for statistical inference and limits our findings' generalizability. Third, the cross-sectional nature of our study design precludes causal inference about the relationships among WVA, gender, and WB. Because temporal ordering between exposure and outcome cannot be established, our ability to explore directionality or mediation pathways is limited. Future research should consider employing longitudinal designs to better capture dynamic interactions and causal processes over time. Future studies should adopt more rigorous sampling designs, such as stratified or matched sampling, or collect data from multiple institutions to achieve a more balanced gender distribution. These strategies would enhance the robustness of interaction effect estimates and improve the external validity of gender-based inferences in studies of WVA and WB.

Furthermore, the observed interaction between gender and alcohol use must be interpreted strictly as exploratory and hypothesis-generating. First, cross-sectional data cannot establish temporal precedence or causality; it is highly plausible that elevated burnout precipitates increased alcohol consumption as a maladaptive coping mechanism (reverse causality), rather than alcohol exposure preceding burnout. Second, the subgroup of daily drinkers was extremely small (*n* = 3), and the male-specific association interval encompassed values near null without adjustment for multiple comparisons. Therefore, our findings should not be construed as indicative of any protective role of alcohol among men, and health interventions should avoid drawing policy inferences from this unverified association prior to longitudinal confirmation.

Finally, the overall response rate of the survey was approximately 40.3% (1,298/3,217), which may introduce potential non-response bias. It is possible that individuals experiencing extreme burnout or heavy workloads were less likely to participate due to time constraints, whereas those with stronger concerns regarding workplace violence might have been more inclined to respond... future multi-center studies with systematic sampling are warranted.

## Conclusion

WVA was identified as a significant independent risk factor for WB among healthcare workers, even after adjusting for relevant occupational and lifestyle variables. Gender moderated this association, with female healthcare workers showing attenuated burnout responses to WVA exposure. Additionally, psychological violence, frequent overtime, irregular shift work, and the nursing profession were independently associated with higher levels of burnout. These findings underscore the multifactorial nature of burnout in healthcare settings and emphasize the importance of targeted interventions that address systemic and role-specific stressors as well as distinct coping mechanisms, such as monitoring maladaptive behaviors in females and providing emotional outlets for males.

## Data Availability

The raw data supporting the conclusions of this article will be made available by the authors, without undue reservation.
